# Clinical and Anatomical Spectrum of Meckel’s Diverticulum: A Systematic Review and Meta-Analysis

**DOI:** 10.3390/jcm15103599

**Published:** 2026-05-08

**Authors:** Dawid Plutecki, Michał Bonczar, Tomasz Kozioł, Grzegorz Fibiger, Mateusz Sporek, Justyna Wajda, Krzysztof Balawender, Jerzy Walocha, Mateusz Koziej, Andrzej Żytkowski, Grzegorz Wysiadecki

**Affiliations:** 1 Department of Anatomy, Jagiellonian University Medical College, 33-332 Kraków, Poland; 2 Youthoria, Youth Research Organization, 30-363 Kraków, Poland; 3 International Evidence-Based Anatomy Working Group, 31-034 Kraków, Poland; 4 Artromed Orthopedic and Rehabilitation Center, 30-059 Kraków, Poland; 5 Department of Normal and Clinical Anatomy, Institute of Medical Sciences, Medical College, Rzeszow University, 35-315 Rzeszów, Poland; 6 Department of Normal and Clinical Anatomy, Faculty of Medicine, University of Social Sciences in Lodz, 90-113 Łódź, Poland; 7 Norbert Barlicki Memorial Teaching Hospital No. 1 of the Medical University of Lodz, 90-153 Łódź, Poland; 8 Laboratory of Neuroanatomy, Department of Normal and Clinical Anatomy, Medical University of Lodz, 90-752 Łódź, Poland

**Keywords:** Meckel’s diverticulum, congenital anomaly, true diverticulum, abdominal anomaly

## Abstract

**Introduction:** Meckel’s diverticulum (MD) is the most common congenital anomaly of the gastrointestinal tract, resulting from incomplete involution of the vitelline duct during the fifth to seventh week of gestation. This study aimed to assess the prevalence, anatomical features, clinical manifestations, and heterotopic tissue of MD through a comprehensive meta-analysis of studies reporting on this anomaly. **Methods:** A systematic search of PubMed, Scopus, ScienceDirect, Web of Science, SciELO, BIOSIS, Current Contents Connect, and the Korean Journal Database was conducted up to March 2024 following PRISMA guidelines. Original studies with extractable data on Meckel’s diverticulum were included, while case reports, reviews, and studies with incomplete data were excluded. Outcomes included prevalence, anatomical features, clinical manifestations, complications, and postoperative outcomes. The study quality was assessed using CATAM and AQUA tools. **Results:** The results of the present meta-analysis comprised 172 studies. The pooled prevalence of MD was 1.56% (95% CI: 0.98–2.28%). Nausea and vomiting were the most frequent symptoms in the pediatric group, with an incidence of 52.34% (95% CI: 38.59–65.92%). In adults, wound infections or dehiscence or anastomotic leakage were the most common postoperative outcomes, with a pooled prevalence of 6.20% (95% CI: 4.02–8.79%). **Conclusions:** This systematic review and meta-analysis provide a comprehensive quantitative synthesis of MD characteristics. Symptomatic cases most frequently presented with intestinal obstruction, diverticulitis, and bleeding, each showing distinct age-related trends. Ectopic gastric mucosa was identified in over 40% of pediatric MD. Postoperative outcomes were generally favorable, particularly in elective settings, with low rates of morbidity and mortality. It is hoped that the findings of this study will aid clinicians in diagnosing, risk-stratifying, and managing patients with MD, particularly in guiding surgical decisions for incidentally discovered cases.

## 1. Introduction

**Figure 1:**
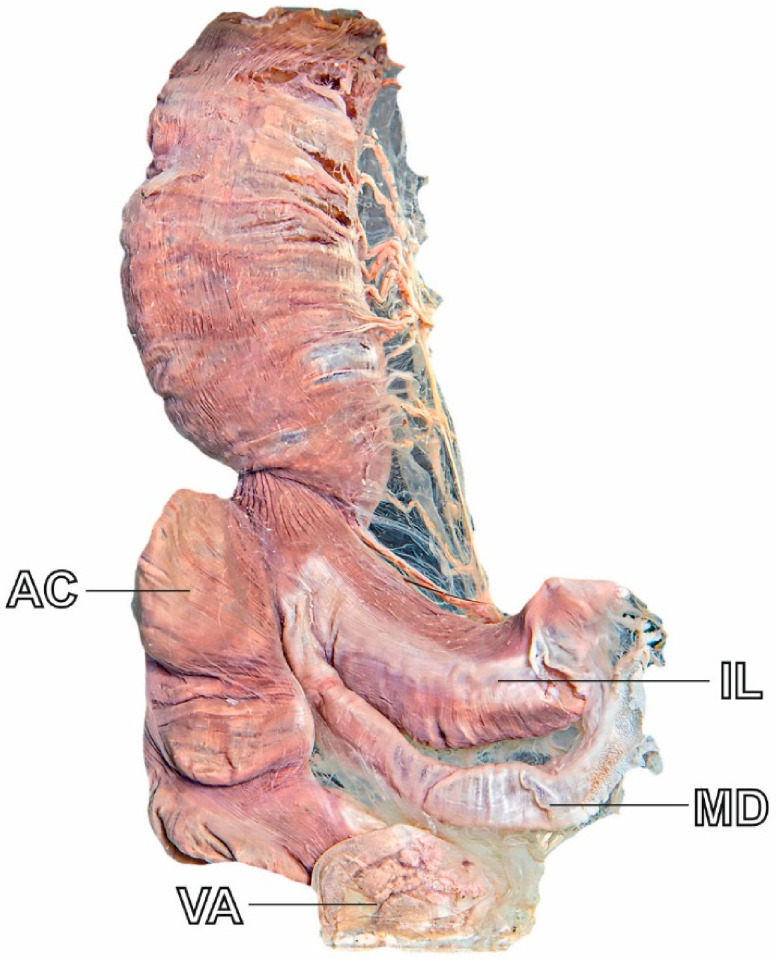
Cadaveric photograph illustrating the anatomical location and gross morphology of MD in relation to the adjacent small intestine. MD—Meckel’s diverticulum; AC—ascending colon; IL—ileum; VA—vermiform appendix.

Meckel’s diverticulum (MD) is the most common congenital anomaly of the gastrointestinal tract. It results from incomplete involution of the vitelline duct during the fifth to seventh week of gestation (Figure 1). MD is a true diverticulum, located on the antimesenteric border of the distal ileum, typically within 60 cm of the ileocecal valve. It contains all layers of the bowel wall [1,2]. Historically described by the “rule of twos,” MD occurs in roughly 2% of the population, 2 inches long, lies 2 feet from the ileocecal valve, and is twice as common in males, though these rules vary [3]. Most MDs remain asymptomatic. However, approximately 4–6% of patients develop complications over their lifetime, most commonly showing bleeding, obstruction, or inflammation [2].

Diagnosis remains challenging due to its nonspecific symptoms and low sensitivity of conventional imaging. Technetium-99 m pertechnetate scintigraphy (“Meckel scan”) can detect ectopic gastric mucosa, especially in pediatric bleeding, but has limitations in adults [4,5,6]. Many cases are diagnosed intraoperatively during evaluation of an acute abdomen or incidentally. While symptomatic MD is universally resected, the management of incidentally discovered, asymptomatic MD is controversial. Some advocate routine removal due to the low operative risk and potential for future complications, while others recommend selective resection based on risk factors such as male sex, diverticulum length > 2 cm, and presence of ectopic tissue [7,8,9].

However, the available literature is highly heterogeneous, with substantial variability in reported incidence, anatomical features, clinical manifestations, and outcomes. Therefore, this systematic review and meta-analysis aims to provide a comprehensive quantitative synthesis of the available evidence on MD.

## 2. Materials and Methods

### 2.1. Search Strategy

Major online medical databases such as PubMed, Scopus, ScienceDirect, Web of Science, SciELO, BIOSIS, Current Contents Connect, and Korean Journal Database were used to gather all relevant studies on Meckel’s diverticulum. The study protocol was not registered prior to the initiation of the study. The study collection ended in March 2024.

The search strategy was developed using a combination of controlled vocabulary terms (e.g., Medical Subject Headings (MeSH)) and free-text keywords related to Meckel’s diverticulum. The following core search terms and their combinations were applied: (“Meckel Diverticulum”[MeSH] OR “Meckel’s diverticulum” OR “Meckel diverticulum”). These terms were further combined with additional keywords depending on the database and scope of analysis, where applicable. Boolean operators (AND, OR) were used to appropriately combine search terms. The search strategy was individually adapted for each database to account for differences in indexing systems and search functionalities. To ensure full reproducibility, the complete search strategies for each database, including exact search strings and field specifications, are provided in Supplementary Table S1.

Neither date, language, article type, nor text availability conditions were applied. An additional search was conducted through the references of the identified studies at the end of the search stage to ensure the accuracy of the process. During the study, the Preferred Reporting Items for Systematic Reviews and Meta-Analyses (PRISMA) guidelines were followed. Furthermore, the Critical Appraisal Tool for Anatomical Meta-analysis (CATAM) and Anatomical Quality Assessment Tool (AQUA) were used to provide the highest quality findings (Supplementary Tables S2 and S3) [10,11,12,13,14]. Potential publication bias was assessed using funnel plots, Doi plots, and the LFK index. The complete set of forest, funnel, and Doi plots and sensitivity analyses for each category is available in Supplementary Material S4. The LFK index was provided in the tables for each analysis. The LFK index should be interpreted as follows: values within ±1 indicate no asymmetry, values between ±1 and ±2 suggest minor asymmetry, and values exceeding ±2 indicate major asymmetry, potentially reflecting publication bias.

### 2.2. Eligibility Assessment

The database search and manual search identified a total of 26,158 studies that were initially evaluated by two independent reviewers. After removing duplicates and irrelevant records, a total of 478 articles were eligible for full-text evaluation. To minimize potential bias and maintain accurate statistical methodology, articles such as case reports, case series, conference reports, reviews, letters to editors, and studies that provided incomplete or irrelevant data were excluded. The inclusion criteria consisted of original studies with extractable numerical data regarding the topic of this study. Finally, a total of 171 studies were included in this meta-analysis [2,15,16,17,18,19,20,21,22,23,24,25,26,27,28,29,30,31,32,33,34,35,36,37,38,39,40,41,42,43,44,45,46,47,48,49,50,51,52,53,54,55,56,57,58,59,60,61,62,63,64,65,66,67,68,69,70,71,72,73,74,75,76,77,78,79,80,81,82,83,84,85,86,87,88,89,90,91,92,93,94,95,96,97,98,99,100,101,102,103,104,105,106,107,108,109,110,111,112,113,114,115,116,117,118,119,120,121,122,123,124,125,126,127,128,129,130,131,132,133,134,135,136,137,138,139,140,141,142,143,144,145,146,147,148,149,150,151,152,153,154,155,156,157,158,159,160,161,162,163,164,165,166,167,168,169,170,171,172,173,174,175,176,177,178,179,180,181,182,183,184]. All included studies were retrospective observational clinical studies based on patient populations undergoing surgical evaluation or resection of MD. Anatomical measurements, when reported, were derived from resected surgical specimens. These methodological characteristics were considered during the data synthesis and interpretation.

### 2.3. Data Extraction

Two independent reviewers extracted data from qualified studies (D.P., T.K.). Qualitative data, such as year of publication, country and continent of origin, data collection methodology, and information on diseases in the studied groups, were collected. Quantitative data, such as sample size, numerical data on anatomical characteristics, morphometry, and relationship with the anatomical surroundings of the MD, were also gathered. Any discrepancies between the studies identified by the two reviewers were resolved by contacting the authors of the original studies, wherever possible, or by consensus with a third reviewer (J.W.).

### 2.4. Outcomes

The outcomes of this study were predefined and categorized as primary and secondary. The primary outcome was the pooled incidence of MD. Secondary outcomes included measures describing its anatomical characteristics (morphometry), clinical presentation, associated complications, postoperative course, and histopathological findings. Specifically, anatomical and morphometric data included the diverticulum diameter and its relationship to the ileocecal valve. Clinical outcomes encompassed symptomatic presentation and its main manifestations. Complication-related outcomes included major disease-associated adverse events, while postoperative outcomes focused on surgical morbidity and mortality. Histopathological outcomes included the presence of heterotopic tissue in MD.

### 2.5. Statistical Analysis

To perform the meta-analyses, MetaXL version 5.3 software (EpiGear International Pty Ltd., Wilston, Queensland, Australia) and Comprehensive Meta-analysis version 3.0 software (Biostat Inc., Englewood, NJ, USA) were used. A random-effects model was used in all analyses. The heterogeneity between the studies was evaluated using both the Chi-squared test and the I-squared statistic [185]. The I-squared statistic was interpreted as follows: 0–40% as “might not be important”; 30–60% as “may represent moderate heterogeneity; 50–90% as “may represent substantial heterogeneity”; 75–100% as “may represent considerable heterogeneity”. A *p*-value of <0.05 and confidence intervals (95% CIs) were used to find statistically significant differences between the studied groups. In cases of overlapping confidence intervals, the differences were considered statistically insignificant.

## 3. Results

### 3.1. Search Results

**Figure 2:**
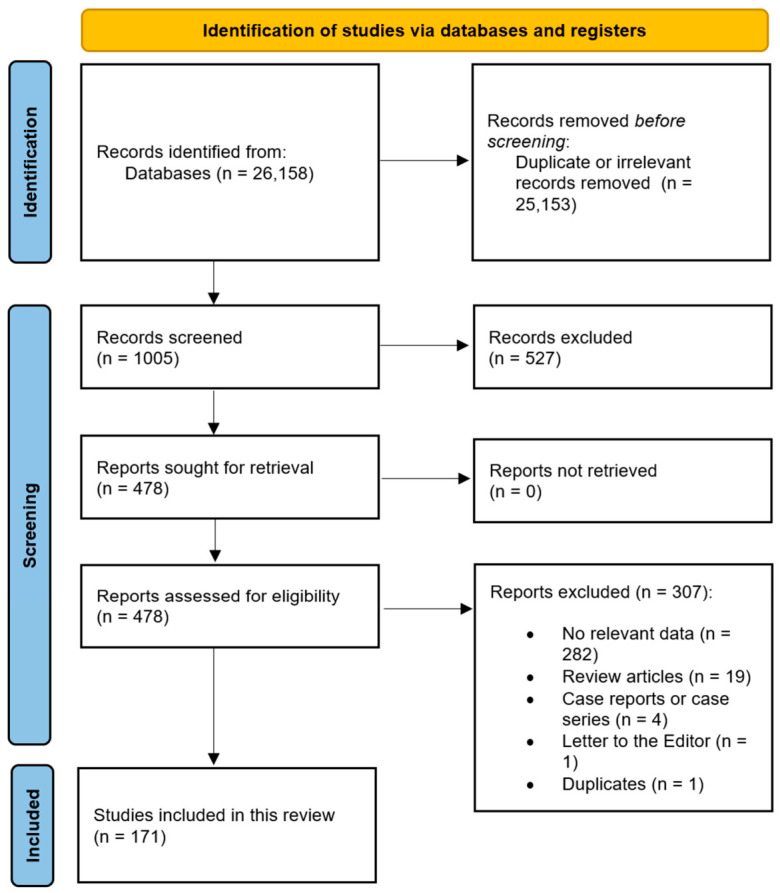
PRISMA flow diagram outlining the study selection process for the meta-analysis, including identification, screening, eligibility assessment, and inclusion of studies.

The overall data collection process is presented in Figure 2. Additionally, the characteristics of all the submitted studies are summarized in Supplementary Table S5. The risk of bias was assessed using the AQUA tool (Supplementary Material S6). The quality of the present meta-analysis was assessed using CATAM tool, which corresponds to a qualitative assessment of “very good” (Supplementary Material S4).

### 3.2. Meckel’s Diverticulum Prevalence

A large pooled cohort of patients was analyzed to assess the prevalence of MD. The findings indicate that MD is an uncommon condition in the general population. Subgroup analyses demonstrated variation across geographic regions, with higher estimates in the Asian population. Male predominance was consistently observed across all analyzed groups. All detailed numerical data are presented in Table 1.

### 3.3. MD Morphometric Parameters

Table 2 presents the detailed morphometric results of MD, such as diameter, length, and distance to the ileocecal valve. Among the analyzed subgroups, the highest mean length of MD was observed in the Asian population.

### 3.4. Clinical Manifestations of Symptomatic MD

**Figure 3:**
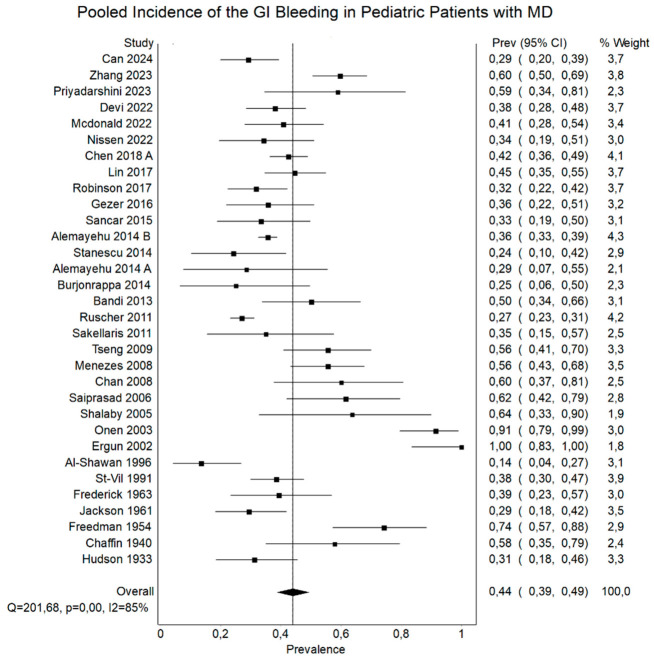
Forest plot regarding the pooled incidence of the gastrointestinal bleeding in pediatric patients with MD, with corresponding 95% confidence intervals.

Table 3 and Figure 3 present the distribution of symptomatic and incidental MD, as well as the clinical manifestations among symptomatic patients. In the overall population, symptomatic and incidental cases occurred with similar frequency. However, clear differences were observed across age groups. Pediatric patients were more likely to present with symptomatic MD, whereas in adults, incidental findings predominated. Regional variation was also noted, with the highest proportion of symptomatic cases observed in Asia, particularly in pediatric populations. Among symptomatic patients, gastrointestinal bleeding was the most common presentation in pediatric populations, while it was less frequent in adults. In contrast, abdominal pain was the predominant symptom in adults and remained common across all groups. Nausea and vomiting were also frequently reported, particularly in pediatric and overall populations.

### 3.5. Complications in Patients with MD

**Figure 4:**
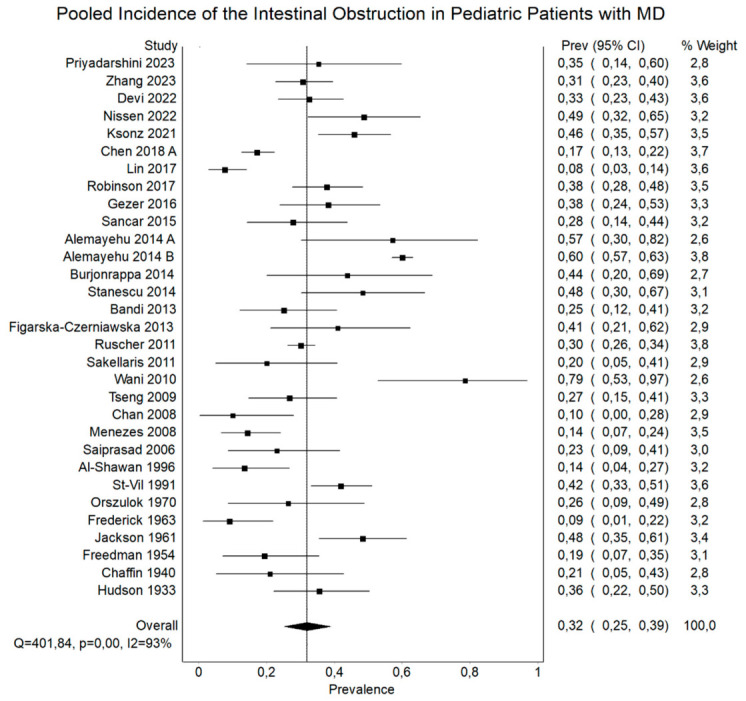
Forest plot regarding the pooled incidence of the intestinal obstruction in pediatric patients with MD, with corresponding 95% confidence intervals.

Table 4 and Figure 4 summarize the complications associated with MD. Intestinal obstruction was the most common complication across all groups, affecting approximately 30% of patients in the overall population. Comparable rates were observed in pediatric and adult subgroups, although higher estimates were noted in Asian populations. Diverticulitis was also frequent, particularly in adults, where it represented one of the leading complications. In contrast, its incidence was lower in pediatric patients. Similarly, perforation occurred more commonly in adults than in children. Intussusception was more prevalent in pediatric populations, whereas it was less frequently observed in adults. Volvulus and Littre’s hernia were relatively uncommon. Peptic ulceration and intestinal gangrene were reported with moderate frequency, while other complications were less common.

### 3.6. Postoperative Outcomes After Removal of the MD

Table 5 summarizes the postoperative outcomes following resection of MD. Overall, postoperative outcomes were favorable, with low rates of mortality and major complications. Mortality remained low across all groups, although slightly higher estimates were observed in pediatric populations. Wound-related complications were among the most commonly reported adverse events and occurred at comparable rates across the age groups. Postoperative intestinal obstruction was also observed, with somewhat higher incidence in pediatric patients compared with adults. Other complications were relatively uncommon. Severe complications such as sepsis were rare but more frequently reported in adult populations.

### 3.7. Heterotopic Tissues Found in MD Histopathology

Table 6 summarizes the distribution of heterotopic tissues in MD. Gastric mucosa was the most frequently identified heterotopic tissue across all groups, with notably higher prevalence in pediatric patients compared with adults. Pancreatic tissue was considerably less common and showed relatively low prevalence across all subgroups, with slightly higher estimates in pediatric populations. Combined gastric and pancreatic heterotopia was observed infrequently overall but appeared more often in pediatric compared with adults. Carcinoid tumors were rare findings and were more commonly reported in adult populations than in pediatric or combined groups. Other heterotopic tissues were uncommon overall but demonstrated higher variability, particularly in pediatric populations.

## 4. Discussion

This meta-analysis provides a comprehensive overview of anatomical, clinical, and histopathological characteristics of MD. By pooling these data, our analysis offers updated, generalizable estimates to inform our understanding of MD epidemiology, clinical presentation, complication risks, and associated outcomes.

Across 4,440,967 individuals, the pooled prevalence of MD was 1.56%, consistent with classical estimates of approximately 2% reported in historical and population-based studies, although prior reports have ranged from 0.3 to 3% depending on the study design and population [23]. Males comprised 65.48% of all cases, with male predominance persisting across subgroups: 73.56% in pediatric populations and 73.86% in adults. The observed male predominance is also consistent with previous studies reporting male proportions ranging from approximately 60–75% among symptomatic patients [43]. Clinically, this pattern highlights young males as a group that warrants closer consideration when MD is discovered incidentally, particularly if additional risk factors, such as diverticulum length greater than 2 cm or evidence of ectopic mucosa, are present [7].

Morphometric pooling demonstrated that the average MD measured 3.64 cm in length, 1.68 cm in diameter, and was located approximately 56.4 cm from the ileocecal valve. These dimensions corroborate the “rule of twos”, with a typically described length between 2 and 5 cm and diameter around 1–2 cm [2]. Notably, prior series associate diverticula > 2 cm with a higher risk of bleeding and obstruction, likely due to an increased likelihood of ectopic mucosa and inversion as a lead point. This reinforces the potential role of morphometric parameters in surgical decision-making.

The differences in clinical presentation between age groups observed in our analysis are also supported by prior literature. Among symptomatic patients, gastrointestinal bleeding was the most commonly reported manifestation in children, occurring in 43.94% (versus 40–50%) of pediatric cases, compared with 18.36% (versus 15–25%) in adults [80]. This likely reflects the higher prevalence of ectopic gastric mucosa in younger patients, which may erode adjacent ileal mucosa and result in painless lower gastrointestinal hemorrhage. In contrast, abdominal pain was the most prevalent symptom in adults, reported in 57.78%, and was similarly common in pediatric patients (49.50%) and mixed cohorts (52.86%). Nausea and vomiting were also frequent, particularly in children (52.34%) and combined groups (40.77%), often signaling bowel obstruction. Other notable findings included abdominal tenderness (43.54%), distention (22.97%), fever (20.28%), and umbilical discharge (9.93%), with the latter specific to pediatric patients and suggestive of a persistent vitelline duct remnant. Preoperative diagnosis remains challenging, with fewer than 10% of symptomatic cases identified before surgery in some series [2]. Our findings underscore the importance of considering MD in obscure gastrointestinal bleeding and unexplained small-bowel obstruction, particularly in young males.

Intestinal obstruction was the most common complication, affecting 30.32% (95% CI, 25.32–35.56%) of symptomatic MD cases, with similar rates in pediatric (32.73%) and adult (38.44%) subgroups. Intussusception occurred in 10.05%, being more common in children (16.61%) than adults (18.84%, though with wide confidence intervals). Volvulus occurred in 7.02%, and Littre’s herniation in 4.83%, both typically presenting as surgical emergencies. Diverticulitis was reported in 30.74%, significantly more frequent in adults (38.18%) than children (17.16%), and often mimicked appendicitis. Perforation was found in 14.49%, with higher rates in adults (24.61%) than pediatric patients (20.03%), possibly reflecting delayed diagnosis or less clinical suspicion in older individuals.

Postoperative outcomes following MD resection were also consistent with prior reports, which generally describe low mortality rates below 2% and complication rates ranging from 5 to 10% [66]. The overall mortality rate was 1.39%, with subgroup estimates of 1.74% in adults and 4.58% in pediatric patients, though the latter showed substantial heterogeneity. Wound complications (including infection, dehiscence, or anastomotic leak) occurred in 4.08%, with similar rates across pediatric (4.18%) and adult (6.20%) cohorts. Postoperative intestinal obstruction was reported in 4.35%, more frequently in children (5.02%) than adults (2.21%), typically due to adhesions or inflammation. Other adverse events, including bleeding (1.96%), abscess formation (1.22%), and pulmonary complications (0.79%), were rare and generally resolved with conservative treatment. Severe complications like sepsis were uncommon, reported in 4.09% of adults and 2.96% of pediatric patients.

Ectopic tissue was identified in a substantial proportion of MD, with 23.41% of pooled specimens containing gastric mucosa. This was notably more frequent in pediatric patients (44.27%) compared with adults (28.51%), aligning with higher bleeding rates in younger cohorts. Pancreatic rests were less common, occurring in 4.45% of cases overall, and were more frequently observed in children (6.14%) than in adults (2.38%). Combined heterotopia (gastric and pancreatic) was observed in 2.81% of cases but increased to 6.88% in pediatric cases. These findings confirm that the gastric mucosa is the predominant ectopic tissue and likely contributes to mucosal ulceration and hemorrhage, especially in children. Carcinoid tumors, the most frequent neoplasms in MD, were identified in 2.34% of pooled cases and in 5.05% of adults. Although rare, these lesions carry significant implications in older patients, often discovered incidentally.

According to the systematic review of epidemiology, presentation, and management of MD in the 21st century by Hansen and Søreide, based on 92 studies, MD is typically located 7 to 200 cm proximal to the ileocecal valve, with an average distance of 52.4 cm [186]. Its length ranges from 0.4 to 11.0 cm (mean 3.05 cm), and its diameter from 0.3 to 7.0 cm (mean 1.58 cm). Only a small proportion of patients (approximately 4% to 9%) become symptomatic. The condition shows a clear male predominance, with a male-to-female ratios ranging from 1.5:1 up to 4:1, and symptomatic cases are most often seen in younger individuals. In pediatric patients, the most common clinical presentations include intestinal obstruction (46.7%), gastrointestinal bleeding (25.3%), and inflammation (19.5%). In adults, obstruction (35.6%), inflammation (29.4%), and bleeding (27.3%) are the leading symptoms. Ectopic tissue is frequently found in symptomatic cases, particularly gastric mucosa, which is present in 24.2% to 71.0% of patients and is strongly associated with bleeding. Less commonly, ectopic pancreatic tissue is identified, occurring in 0% to 12.0% of cases [2]. MD is challenging to diagnose on account of its clinical presentations. Patients with symptomatic MD often undergo radiography of the abdomen to exclude obstruction or perforation in the emergency department. Occasionally a large gas-filled diverticulum may be visible as a round or oval-shaped radiolucency, which may occur when the narrowed neck of the diverticulum acts as a ball-valve and entraps the intestinal gas within its lumen or that food and other objects may serve as a nidus for development of radiopaque or laminated enteroliths [187]. Technetium-99 m pertechnetate scintigraphy (“Meckel scan”) is still a first-line modality, especially in pediatric patients presenting gastrointestinal bleeding. In this group, the reported sensitivity ranging from approximately 80 to 94% and specificity reaching 95–97% with a diameter over 1 cm [116,188,189]. However, this accuracy decreases substantially in adults, where the sensitivity drops to around 60% and the specificity becomes significantly lower [110]. The typical indications of a “Meckel scan” are localization of a MD with functioning gastric mucosa, and explanation of blood in feces, abdominal pain, bleeding, diverticulitis, or intestinal obstruction [190]. Among 94 patients examined by Li et al. in 2025, pertechnetate scintigraphy demonstrated a sensitivity of 76.9% and specificity of 100%, with an overall diagnostic accuracy of 93.6% [191]. False-negative results were observed in a small proportion of cases, highlighting limitations of the method [191]. However, the other diagnostic modalities are not specific to MD and are based on data from non-MD populations. Computed tomography (CT) is frequently used, but the identification of MD is limited, with the sensitivity generally reported between 30 and 50%. On CT imaging, a small MD can be difficult to differentiate from normal small bowel loops. However, when it exceeds 3 cm in diameter, it typically appears as a blind-ending structure filled with gas or fluid and may also contain foreign bodies or enteroliths [189].

Its main value lies in detecting complications such as obstruction, inflammation, or perforation [116,192]. Le Nguyen et al. reported two cases of complicated MD in adults presenting atypical epigastric pain with fever (Case 1) and right flank pain with diarrhea (Case 2) and diagnosed with diverticulitis via abdominal ultrasonography and CT imaging instead of initial appendicitis, as confirmed through laparoscopic exploration [193]. Endoscopic techniques have also gained importance, particularly in patients with obscure gastrointestinal bleeding. Capsule endoscopy offers a diagnostic yield of approximately 50–70%, although these estimates are derived from studies on small bowel bleeding [194]. Based on a review of 33 publications (44 cases), the most frequent finding on capsule endoscopy was the double lumen sign. Moreover, patients had an average of three negative investigations performed before their endoscopy [186]. Similarly, balloon-assisted enteroscopy provides a higher sensitivity, estimated at 80–90% in selected cases, and allows for both diagnosis and therapeutic intervention [194]. Angiography remains an option in cases of active bleeding, with sensitivity ranging from 40 to 86%, depending largely on the bleeding rate (typically ≥ 0.3–0.5 mL/min) [195]. The vascular supply of MD typically arises from branches of the ileocolic artery, itself a branch of the superior mesenteric artery. In some cases, a persistent vitelline artery is also present—an elongated vessel originating from a distal ileal branch that traverses the ileum to reach and supply the diverticulum along its antimesenteric border [187,189,196]. Overall, the wide variability in diagnostic performance across modalities and patient populations, along with inconsistent reporting in the literature, makes it difficult to draw firm conclusions or perform a reliable quantitative synthesis. This highlights an important limitation of the current evidence base and underscores the need for more standardized diagnostic strategies in future studies.

This study has limitations that warrant acknowledgment. The study protocol was not registered prior to the initiation of the study. Furthermore, potential bias may be present due to the varying accuracy of data extracted from diverse publications, which, in turn, affects the reliability of the results obtained in this meta-analysis. All included studies were retrospective in design, which may introduce selection and reporting bias inherent to observational data. Second, anatomical parameters such as diverticulum length and diameter were measured in resected surgical specimens, which may not fully represent the in vivo anatomy of MD prior to surgical manipulation. Moreover, morphometric analysis of the MD in relation to the height of subjects or other parameters was not feasible due to the lack of such information in the primary studies. This should be considered when interpreting morphometric results. Third, variability across studies in patient populations, indications for surgery, and diagnostic pathways contribute to heterogeneity in the pooled estimates. Although a random-effects model was applied, residual methodological variability cannot be excluded. Furthermore, most of the included studies originate from Asia, Europe, and North America, which potentially limits the generalizability of the findings. Notably, although geographic subgroup analyses were conducted in an attempt to reduce data the heterogeneity across multiple categories, the results remained heterogeneous in some categories. This may reflect the true variability of these characteristics within the population or may be due to a limited sample size; therefore, those findings should be interpreted with caution. Lastly, due to the insufficient amount of consistent data available in the literature and inconsistent reporting across the included studies, a quantitative synthesis of diagnostic pathways for MD could not be performed, which limits the ability to systematically evaluate differences in diagnostic approaches. Many of the obtained LFK index values indicate major asymmetry; therefore, these findings should also be interpreted with caution, as substantial publication bias may be present. Moreover, these results highlight specific categories in which the existing literature remains limited and may require further refinement and standardization. Despite these limitations, our meta-analysis aims to provide insights into the anatomy, morphology, and clinical manifestations of the MD based on evidence from the literature that meets the criteria of evidence-based anatomy.

## 5. Conclusions

This systematic review and meta-analysis provide a comprehensive quantitative synthesis of MD characteristics. The pooled prevalence of MD was 1.56%, with a consistent male predominance across all age groups. Symptomatic cases most frequently presented with intestinal obstruction, diverticulitis, and bleeding, each showing distinct age-related trends. Ectopic gastric mucosa was identified in over 40% of pediatric population. Postoperative outcomes were generally favorable, particularly in elective settings, with low rates of morbidity and mortality. Overall, the results provide a useful overview of the epidemiology and clinical features of MD, but their applicability to individual patient management may be limited due to the substantial heterogeneity between included studies. Further well-designed prospective studies are needed to better define optimal diagnostic and therapeutic strategies.

## Figures and Tables

**Table 1:** Statistical results of this meta-analysis regarding the pooled incidence and sex distribution among patients diagnosed with MD. LCI—lower confidence interval; HCI—higher confidence interval; Q—Cochran’s Q; I^2^—I-squared; LFK—LFK index.

Group			N	Pooled Incidence	LCI	HCI	Q	I^2^	LFK
General Incidence									
Overall			4,440,967	1.56%	0.98%	2.28%	2996.20	99.30	9.70
Asia			17,536	2.25%	1.04%	3.89%	240.27	97.09	2.89
Europe			66,205	1.56%	0.71%	2.73%	711.80	98.88	3.63
North America			4,356,026	0.32%	0.05%	0.78%	132.09	97.73	9.82
Males vs. Females									
Adults	Overall	Males	1410	73.86%	68.24%	79.12%	78.74	75.87	1.04
		Females		26.14%	20.88%	31.76%	78.74	75.87	−1.05
	Asia	Males	415	76.31%	67.63%	84.03%	22.31	68.62	−0.80
		Females		23.69%	15.97%	32.37%	22.31	68.62	0.79
	Europe	Males	208	71.70%	62.75%	79.87%	7.40	45.95	1.70
		Females		28.30%	20.13%	37.25%	7.40	45.95	−1.71
	North America	Males	575	68.99%	37.77%	93.63%	33.16	93.97	0.26
		Females		31.01%	6.37%	62.23%	33.16	93.97	−0.27
Overall	Overall	Males	7890	65.48%	62.34%	68.56%	270.91	83.76	−1.90
		Females		34.52%	31.44%	37.66%	270.91	83.76	1.90
	Asia	Males	4071	71.20%	69.03%	73.33%	14.90	19.48	−0.35
		Females		28.80%	26.67%	30.97%	14.90	19.48	0.35
	Europe	Males	1564	62.36%	57.34%	67.24%	56.09	73.26	1.23
		Females		37.64%	32.76%	42.66%	56.09	73.26	−1.24
	North America	Males	2164	63.47%	55.57%	71.03%	130.07	90.01	−0.74
		Females		36.53%	28.97%	44.43%	130.07	90.01	0.74
Pediatric	Overall	Males	4787	73.56%	71.08%	75.96%	162.54	66.16	0.04
		Females		26.44%	24.04%	28.92%	162.54	66.16	−0.04
	Asia	Males	1806	72.94%	68.49%	77.17%	108.27	75.06	0.13
		Females		27.06%	22.83%	31.51%	108.27	75.06	−0.13
	Europe	Males	641	76.47%	71.07%	81.47%	22.84	56.22	0.70
		Females		23.53%	18.53%	28.93%	22.84	56.22	−0.71
	North America	Males	2208	73.18%	69.53%	76.68%	25.61	53.14	−0.27
		Females		26.82%	23.32%	30.47%	25.61	53.14	0.26

**Table 2:** Statistical results of this meta-analysis regarding the morphometric parameters of the MD. All values are presented in centimeters (cm).

Group		N	Mean	Standard Error	Variance	Lower Limit	Upper Limit	Z-Value	*p*-Value
Diameter									
Adults	Overall	449	1.93	0.14	0.02	1.65	2.21	13.70	0.00
	Asia	158	1.77	0.22	0.05	1.33	2.21	7.86	0.00
	Europe	131	2.09	0.30	0.09	1.51	2.68	6.98	0.00
Overall	Overall	135	1.68	0.09	0.01	1.51	1.85	19.36	0.00
	Europe	95	1.66	0.14	0.02	1.39	1.94	11.87	0.00
Pediatric	Overall	695	1.62	0.10	0.01	1.43	1.81	16.58	0.00
	Asia	399	1.62	0.13	0.02	1.37	1.87	12.91	0.00
	Europe	296	1.62	0.18	0.03	1.27	1.97	8.98	0.00
Length									
Adults	Overall	463	3.66	0.34	0.11	3.00	4.33	10.83	0.00
	Asia	172	3.82	0.65	0.42	2.55	5.08	5.90	0.00
	Europe	131	3.36	0.19	0.04	2.97	3.74	17.21	0.00
Overall	Overall	258	3.64	0.45	0.20	2.77	4.52	8.18	0.00
	Asia	123	4.25	0.83	0.69	2.62	5.89	5.11	0.00
	Europe	95	2.50	0.46	0.21	1.60	3.40	5.43	0.00
Pediatric	Overall	695	3.28	0.28	0.08	2.74	3.82	11.89	0.00
	Asia	399	3.52	0.41	0.16	2.72	4.31	8.68	0.00
	Europe	296	2.71	0.08	0.01	2.56	2.87	34.98	0.00
Distance to the Ileocecal Valve									
Adults	Overall	151	60.79	1.15	1.32	58.55	63.04	53.01	0.00
Overall	Overall	245	56.40	3.69	13.61	49.17	63.62	15.29	0.00
	Asia	88	63.33	7.68	58.97	48.28	78.38	8.25	0.00
	Europe	143	48.51	2.56	6.56	43.49	53.53	18.94	0.00
Pediatric	Overall	775	48.51	2.01	4.06	44.56	52.46	24.08	0.00
	Asia	625	45.67	1.91	3.65	41.92	49.41	23.91	0.00
	Europe	150	55.24	2.02	4.09	51.28	59.21	27.32	0.00

**Table 3:** Statistical results of this meta-analysis regarding the pooled incidence of the symptomatic and the associated clinical manifestations among patients diagnosed with MD. LCI—lower confidence interval; HCI—higher confidence interval; Q—Cochran’s Q; I^2^—I-squared; LFK—LFK index.

Group			N	Pooled Incidence	LCI	HCI	Q	I^2^	LFK
Symptomatic vs. Incidental MD									
Adults	Overall	Symptomatic	2043	39.79%	31.28%	48.61%	264.83	92.83	3.66
		Incidental		60.21%	51.39%	68.72%	264.83	92.83	−3.66
	Asia	Symptomatic	228	35.65%	14.50%	59.88%	19.29	89.63	−0.55
		Incidental		64.35%	40.12%	85.50%	19.29	89.63	0.54
	Europe	Symptomatic	552	42.61%	32.33%	53.21%	47.38	83.11	0.46
		Incidental		57.39%	46.79%	67.67%	47.38	83.11	−0.47
	North America	Symptomatic	1076	27.77%	15.11%	42.43%	74.93	93.33	2.60
		Incidental		72.23%	57.57%	84.89%	74.93	93.33	−2.61
Overall	Overall	Symptomatic	6627	49.57%	41.95%	57.20%	1159.65	96.81	1.64
		Incidental		50.43%	42.80%	58.05%	1159.65	96.81	−1.64
	Asia	Symptomatic	2814	65.00%	51.90%	77.08%	100.49	92.04	4.53
		Incidental		35.00%	22.92%	48.10%	100.49	92.04	−4.53
	Europe	Symptomatic	1352	43.02%	32.17%	54.22%	210.64	93.83	0.82
		Incidental		56.98%	45.78%	67.83%	210.64	93.83	−0.82
	North America	Symptomatic	2384	46.84%	30.86%	63.13%	521.48	97.51	6.72
		Incidental		53.16%	36.87%	69.14%	521.48	97.51	−6.72
Pediatric	Overall	Symptomatic	3653	65.91%	59.44%	72.09%	526.82	93.55	0.48
		Incidental		34.09%	27.91%	40.56%	526.82	93.55	−0.48
	Asia	Symptomatic	1395	75.57%	65.49%	84.42%	226.73	93.83	0.24
		Incidental		24.43%	15.58%	34.51%	226.73	93.83	−0.24
	Europe	Symptomatic	839	55.02%	44.55%	65.27%	90.36	88.93	1.67
		Incidental		44.98%	34.73%	55.45%	90.36	88.93	−1.67
	North America	Symptomatic	1387	65.33%	56.92%	73.30%	48.84	85.67	1.93
		Incidental		34.67%	26.70%	43.08%	48.84	85.67	−1.93
Clinical Manifestations of the Symptomatic MD									
Gastrointestinal Bleeding	Adults	Overall	611	22.29%	11.04%	35.91%	159.69	91.86	1.34
		Asia	185	30.06%	0.00%	73.01%	113.27	96.47	2.94
		Europe	137	16.71%	9.11%	25.95%	6.63	39.71	0.86
		North America	175	36.92%	27.50%	46.84%	1.14	12.54	−4.44
	Overall	Overall	2819	26.95%	20.73%	33.65%	320.05	91.25	1.97
		Asia	1445	38.66%	20.41%	58.59%	118.99	94.12	5.40
		Europe	549	20.33%	9.48%	33.77%	103.88	91.34	1.53
		North America	792	24.81%	15.20%	35.82%	85.28	89.45	−0.04
	Pediatric	Overall	3059	43.94%	38.75%	49.20%	201.68	84.63	2.17
		Asia	959	48.74%	38.88%	58.65%	121.90	88.51	1.51
		Europe	241	38.61%	28.31%	49.43%	14.05	64.40	0.22
		North America	1848	37.71%	31.74%	43.87%	41.01	75.62	1.83
Abdominal Pain	Adults	Overall	354	59.32%	38.25%	78.86%	106.20	92.47	−0.43
		Asia	85	36.73%	8.98%	69.46%	5.00	80.02	−3.36
		Europe	93	38.60%	1.50%	84.82%	30.27	93.39	−0.15
		North America	25	86.72%	45.22%	100.00%	5.53	81.93	1.74
	Overall	Overall	600	54.35%	40.52%	67.86%	153.34	90.87	−1.52
		Asia	148	45.46%	12.09%	80.92%	65.80	93.92	−2.37
		Europe	172	60.50%	27.25%	89.66%	46.42	93.54	−1.16
		North America	280	57.11%	39.31%	74.07%	37.93	86.82	−1.02
	Pediatric	Overall	820	49.50%	34.95%	64.09%	275.24	93.82	−0.11
		Asia	406	57.92%	38.12%	76.55%	67.93	91.17	−0.42
		Europe	135	57.74%	20.55%	91.09%	47.85	93.73	0.73
		North America	268	37.61%	10.85%	68.57%	102.98	95.14	3.21
Nausea/Vomiting	Overall	Overall	211	40.77%	21.01%	62.09%	41.99	88.09	1.56
		Asia	104	24.91%	6.97%	48.24%	10.21	80.41	−0.95
		North America	38	64.68%	16.41%	100.00%	8.16	87.75	−1.91
	Pediatric	Overall	555	52.34%	38.59%	65.92%	96.75	88.63	0.84
		Asia	386	62.34%	38.25%	83.82%	75.95	93.42	2.77
		Europe	94	46.84%	36.84%	56.98%	0.12	0.00	−2.19
		North America	64	49.12%	21.28%	77.22%	9.63	79.24	−0.22
Abdominal Tenderness	Overall	Overall	229	43.54%	29.32%	58.30%	16.27	75.42	2.70
		Asia	57	46.96%	32.92%	61.25%	1.10	9.44	−3.11
		North America	157	33.13%	20.34%	47.28%	2.94	66.04	2.43
Abdominal Distention	Pediatric	Overall	330	22.97%	11.60%	36.64%	19.69	74.60	−0.18
		Asia	286	22.53%	17.87%	27.56%	0.70	0.00	−1.27
		North America	33	34.67%	0.00%	100.00%	17.15	94.17	1.97
Fever	Overall	Overall	180	19.30%	5.62%	37.75%	16.15	81.42	3.09
		Asia	57	36.12%	4.60%	74.96%	5.22	80.85	3.08
		North America	119	9.24%	4.68%	15.06%	0.05	0.00	−3.69
Umbilical Discharge	Pediatric	Overall	157	9.93%	4.44%	17.15%	5.00	39.97	−1.67
		Europe	99	13.46%	7.38%	20.95%	0.15	0.00	−2.28

**Table 4:** Statistical results of this meta-analysis regarding the pooled incidence of the complications in patients with MD. LCI—lower confidence interval; HCI—higher confidence interval; Q—Cochran’s Q; I^2^—I-squared; LFK—LFK index.

Group			N	Pooled Incidence	LCI	HCI	Q	I^2^	LFK
Complications in Patients with MD									
Intestinal Obstruction	Adults	Overall	618	38.44%	26.72%	50.86%	119.68	88.30	1.19
		Asia	182	52.12%	23.88%	79.74%	48.42	91.74	0.80
		Europe	137	30.24%	22.86%	38.16%	3.02	0.00	2.06
		North America	185	18.16%	11.92%	25.34%	2.14	6.71	1.35
	Overall	Overall	2783	30.32%	25.32%	35.56%	180.31	83.36	−2.15
		Asia	1364	40.23%	29.57%	51.36%	16.79	70.23	−2.40
		Europe	580	28.38%	23.68%	33.33%	16.69	34.07	1.12
		North America	839	27.32%	21.04%	34.08%	41.59	71.15	−0.73
	Pediatric	Overall	3001	32.73%	26.09%	39.72%	414.39	92.52	−1.67
		Asia	842	29.00%	20.05%	38.83%	98.24	87.79	0.97
		Europe	365	35.26%	25.47%	45.70%	31.76	74.81	−2.16
		North America	1794	35.02%	23.31%	47.70%	172.80	94.79	−4.76
Intussusception	Adults	Overall	329	18.84%	5.20%	37.45%	63.43	90.54	2.55
		Asia	126	27.27%	0.00%	67.24%	49.61	93.95	1.70
		North America	173	10.09%	5.10%	16.46%	1.08	7.49	4.58
	Overall	Overall	2432	10.05%	7.81%	12.53%	53.30	53.10	2.10
		Asia	1310	10.30%	8.71%	12.00%	0.95	0.00	7.16
		Europe	605	10.43%	6.50%	15.13%	31.90	62.39	1.53
		North America	484	7.58%	4.36%	11.56%	11.04	36.61	3.75
	Pediatric	Overall	1882	16.61%	12.74%	20.88%	119.60	79.10	−0.47
		Asia	822	14.09%	6.99%	23.03%	99.59	89.96	−0.09
		Europe	316	19.24%	13.65%	25.53%	10.42	42.40	0.43
		North America	733	18.48%	15.76%	21.37%	2.30	0.00	−2.08
Diverticulitis	Adults	Overall	620	42.31%	29.35%	55.81%	131.68	90.13	2.91
		Asia	236	49.31%	23.38%	75.41%	87.14	93.11	0.37
		Europe	96	38.20%	21.78%	56.04%	5.37	62.78	−0.17
		North America	174	38.81%	0.00%	91.31%	11.74	91.48	4.50
	Overall	Overall	1538	30.74%	26.19%	35.48%	97.57	70.28	1.87
		Asia	85	44.30%	19.02%	71.08%	10.38	80.73	0.81
		Europe	646	30.95%	23.53%	38.88%	56.79	75.35	2.81
		North America	774	26.79%	21.57%	32.36%	21.42	53.31	0.82
	Pediatric	Overall	1885	17.16%	12.32%	22.60%	146.18	83.58	3.18
		Asia	544	23.17%	13.01%	35.13%	87.01	88.51	1.81
		Europe	260	11.00%	5.83%	17.47%	12.98	53.78	2.07
		North America	1070	8.96%	7.32%	10.75%	4.91	0.00	5.78
Perforation	Adults	Overall	265	24.61%	13.65%	37.46%	22.06	77.34	−0.21
		Asia	99	30.57%	3.90%	65.67%	15.07	86.73	2.05
	Overall	Overall	1406	14.49%	11.36%	17.92%	59.26	61.19	2.39
		Asia	135	21.41%	14.91%	28.70%	1.11	0.00	1.43
		Europe	595	14.13%	9.30%	19.74%	35.24	65.95	1.83
		North America	676	12.10%	7.84%	17.12%	14.37	65.20	1.48
	Pediatric	Overall	807	20.03%	12.24%	29.12%	121.05	87.61	1.75
		Asia	565	26.31%	12.35%	43.04%	114.73	93.03	2.83
		Europe	147	11.21%	5.42%	18.63%	4.61	34.97	−1.99
		North America	95	15.41%	8.80%	23.39%	0.06	0.00	0.53
Littres Herniation	Adults	Overall	118	8.51%	0.57%	22.22%	8.94	66.45	1.34
		Europe	37	17.01%	6.41%	30.93%	0.00	0.00	3.21
	Overall	Overall	617	4.83%	2.02%	8.68%	20.73	61.42	4.80
		Europe	367	4.48%	2.22%	7.42%	7.48	19.78	4.26
		North America	250	8.84%	0.00%	43.90%	9.31	89.26	4.89
	Pediatric	Overall	288	3.52%	0.00%	9.39%	5.41	63.06	4.62
		Asia	253	4.12%	0.00%	16.59%	4.62	78.37	4.35
Peptic Ulcer	Adults	Overall	125	29.88%	22.17%	38.19%	0.69	0.00	−4.10
	Overall	Overall	410	23.00%	12.48%	35.46%	63.38	85.80	0.42
		Europe	233	25.19%	6.94%	48.91%	60.84	91.78	−0.72
		North America	149	20.26%	14.07%	27.23%	2.03	1.56	−1.50
	Pediatric	Overall	266	29.00%	20.46%	38.34%	15.13	60.33	−0.25
		Asia	62	22.61%	0.00%	58.02%	8.35	88.02	−2.03
		Europe	140	34.14%	23.55%	45.56%	3.57	43.91	2.61
		North America	64	25.44%	12.82%	40.45%	1.49	33.05	−2.61
Volvulus	Overall	Overall	2071	7.02%	4.47%	10.07%	39.74	69.81	0.44
		Europe	433	6.51%	1.88%	13.32%	28.38	78.86	3.40
		North America	378	6.70%	3.08%	11.51%	5.26	42.99	3.40
	Pediatric	Overall	581	13.75%	5.97%	23.87%	55.87	87.47	2.16
		Asia	410	15.53%	3.16%	33.55%	51.23	92.19	2.75
		North America	136	13.48%	5.21%	24.52%	1.63	38.79	−3.68
Intestinal Gangrene	Overall	Overall	247	11.36%	4.70%	20.23%	18.08	66.82	2.97
		Europe	77	17.58%	6.71%	31.79%	5.84	48.61	1.36
		North America	128	3.37%	0.81%	7.34%	0.40	0.00	3.38
	Pediatric	Overall	90	12.75%	6.60%	20.45%	2.96	0.00	3.63
		Asia	40	12.87%	2.38%	28.65%	1.43	30.14	2.30
Anemia	Overall	Overall	108	4.22%	1.10%	8.96%	1.07	0.00	4.03
	Pediatric	Overall	440	16.40%	0.00%	42.02%	124.80	96.79	1.04
		Asia	409	20.23%	0.00%	53.33%	119.48	97.49	1.92
Internal Herniation	Overall	Overall	143	15.99%	9.43%	23.83%	3.85	22.15	−0.77
		Europe	108	18.02%	8.17%	30.43%	2.07	51.63	−2.47
		North America	35	9.59%	1.68%	21.87%	0.02	0.00	−2.11
Fistula	Pediatric	Overall	141	11.59%	6.63%	17.66%	3.19	6.06	−1.64
		North America	83	9.72%	3.39%	18.54%	2.66	24.88	−0.73

**Table 5:** Statistical results of this meta-analysis regarding the pooled incidence of the postoperative outcomes after removal of the MD. LCI—lower confidence interval; HCI—higher confidence interval; Q—Cochran’s Q; I^2^—I-squared; LFK—LFK index.

Group			N	Pooled Incidence	LCI	HCI	Q	I^2^	LFK
	Postoperative Outcomes								
Death	Adults	Overall	1429	1.74%	0.58%	3.44%	39.14	66.78	3.04
		Asia	241	4.40%	0.96%	9.76%	5.66	47.03	1.09
		Europe	250	1.35%	0.22%	3.24%	2.15	0.00	−0.86
		North America	753	0.41%	0.00%	1.63%	6.24	67.97	3.38
	Overall	Overall	5773	1.39%	0.73%	2.24%	72.35	69.59	3.35
		Asia	2631	1.37%	0.96%	1.86%	2.53	0.00	−6.43
		Europe	655	1.24%	0.31%	2.69%	9.11	34.13	3.90
		North America	2473	1.80%	0.52%	3.74%	50.25	80.10	5.99
	Pediatric	Overall	2141	4.58%	2.35%	7.48%	122.21	84.45	3.52
		Asia	761	1.72%	0.44%	3.70%	15.85	55.84	3.84
		Europe	236	5.34%	0.00%	16.81%	21.09	85.78	1.62
		North America	1144	7.81%	2.28%	15.88%	76.84	90.89	4.61
Wound Infections/Dehiscence/Anastomotic Leakage	Adults	Overall	804	6.20%	4.02%	8.79%	7.86	23.69	4.30
		Europe	222	5.03%	1.76%	9.70%	3.27	38.86	−1.45
		North America	553	5.57%	3.38%	8.24%	1.11	10.24	4.28
	Overall	Overall	3128	4.08%	2.24%	6.41%	99.03	83.84	4.03
		Asia	217	4.06%	1.78%	7.14%	0.26	0.00	0.79
		Europe	776	4.60%	2.32%	7.57%	15.95	62.38	0.30
		North America	2121	2.94%	0.54%	6.82%	46.33	89.21	6.47
	Pediatric	Overall	1875	4.18%	2.27%	6.61%	46.04	76.11	3.43
		Asia	928	5.65%	2.40%	10.05%	40.53	80.26	4.52
		North America	974	2.31%	0.69%	4.75%	5.47	63.47	−4.98
Intestinal Obstruction	Adults	Overall	431	2.21%	0.95%	3.93%	3.22	6.70	3.03
		Asia	152	3.18%	0.00%	9.48%	1.66	39.82	4.08
	Overall	Overall	2667	4.35%	1.87%	7.73%	98.75	88.86	4.50
		Europe	703	5.24%	1.98%	9.80%	22.90	78.17	3.59
		North America	1865	2.38%	0.00%	6.76%	39.60	92.42	4.19
	Pediatric	Overall	1422	5.02%	2.80%	7.81%	54.85	76.30	2.50
		Asia	989	6.17%	2.72%	10.81%	48.59	83.53	3.70
		Europe	167	3.26%	1.01%	6.59%	1.71	0.00	2.08
		North America	266	2.41%	0.48%	5.52%	1.63	38.78	−2.19
Bleeding	Adults	Overall	691	1.57%	0.76%	2.64%	0.26	0.00	0.25
		Europe	185	1.28%	0.07%	3.54%	0.24	0.00	2.28
	Overall	Overall	2002	1.96%	0.18%	5.12%	32.48	84.61	7.22
		Europe	223	2.90%	0.00%	8.71%	2.71	63.15	3.51
		North America	1765	1.49%	0.00%	4.92%	20.54	90.26	5.10
	Pediatric	Overall	813	4.85%	3.47%	6.44%	0.00	0.00	−3.50
Abscess	Overall	Overall	1835	1.22%	0.00%	3.31%	15.71	80.90	5.48
		North America	1769	0.61%	0.00%	1.87%	6.97	71.31	5.81
	Pediatric	Overall	315	2.06%	0.03%	6.09%	6.24	67.93	1.86
		Asia	213	3.33%	0.00%	10.78%	5.73	82.55	2.33
Pulmonary Complications	Overall	Overall	1854	0.79%	0.00%	2.10%	9.88	69.65	6.09
		Europe	237	0.92%	0.00%	2.87%	1.10	8.98	3.13
		North America	1617	0.55%	0.00%	2.47%	5.27	81.02	4.26
	Pediatric	Overall	871	2.61%	0.00%	7.42%	20.43	85.32	4.74
		Asia	108	6.69%	2.63%	12.27%	0.82	0.00	−2.52
Sepsis	Adults	Overall	643	4.09%	1.14%	8.53%	3.69	72.91	−3.12
	Pediatric	Overall	795	2.96%	0.00%	8.42%	11.22	82.17	4.65
		Asia	114	5.47%	1.93%	10.52%	0.74	0.00	−2.34

**Table 6:** Statistical results of this meta-analysis regarding the pooled incidence of heterotopic tissues found in histopathological evaluation after resection of the MD. LCI—lower confidence interval; HCI—higher confidence interval; Q—Cochran’s Q; I^2^—I-squared; LFK—LFK index.

Group			N	Pooled Incidence	LCI	HCI	Q	I^2^	LFK
Heterotopic Tissues									
Gastric Mucosa	Adults	Overall	1326	28.51%	17.91%	40.40%	332.31	94.88	0.14
		Asia	500	30.12%	7.66%	58.32%	240.85	97.09	−0.70
		Europe	303	19.70%	14.73%	25.18%	5.02	20.31	0.18
		North America	110	38.23%	0.00%	100.00%	44.67	97.76	−1.99
	Overall	Overall	4320	23.41%	19.12%	28.00%	389.79	89.99	3.39
		Asia	1100	27.85%	17.09%	40.02%	98.57	91.88	4.72
		Europe	1536	24.99%	17.14%	33.75%	201.23	92.55	1.53
		North America	1607	18.17%	13.19%	23.75%	45.53	71.45	4.03
	Pediatric	Overall	3306	44.27%	38.16%	50.47%	660.49	91.98	0.94
		Asia	1612	42.97%	34.53%	51.62%	285.52	91.24	0.02
		Europe	956	38.97%	28.30%	50.19%	162.06	91.36	3.75
		North America	643	46.87%	32.31%	61.70%	114.38	92.13	1.14
Pancreatic Tissue	Adults	Overall	876	2.38%	1.46%	3.50%	8.96	0.00	2.30
		Asia	471	3.04%	1.35%	5.33%	7.39	32.34	2.11
		Europe	184	1.28%	0.07%	3.56%	0.25	0.00	2.30
	Overall	Overall	3487	4.45%	2.74%	6.54%	131.73	82.54	1.94
		Asia	853	4.40%	2.28%	7.14%	9.40	36.17	4.63
		Europe	1193	4.36%	1.00%	9.57%	114.87	91.29	2.15
		North America	1364	3.78%	2.02%	6.04%	6.56	39.04	4.16
	Pediatric	Overall	2096	6.14%	4.18%	8.43%	106.11	72.67	2.56
		Asia	1080	7.39%	3.50%	12.48%	87.40	85.13	2.74
		Europe	539	5.25%	3.41%	7.44%	9.86	8.74	1.31
		North America	415	3.27%	1.33%	5.97%	4.60	34.71	1.95
Gastric + Pancreatic Tissue	Adults	Overall	461	3.50%	1.65%	5.97%	6.01	33.39	2.97
		North America	110	5.09%	0.87%	11.91%	1.62	38.21	−1.99
	Overall	Overall	1510	2.81%	1.53%	4.43%	22.76	51.67	4.65
		Asia	676	4.19%	0.00%	11.36%	9.50	78.95	5.99
		Europe	533	2.06%	1.01%	3.47%	1.33	0.00	0.94
		North America	224	5.45%	1.10%	12.27%	6.55	54.18	4.76
	Pediatric	Overall	2061	6.88%	5.26%	8.70%	60.10	51.75	2.62
		Asia	1324	7.43%	5.02%	10.25%	48.04	64.61	2.82
		Europe	340	5.40%	2.75%	8.81%	6.84	26.87	1.86
		North America	335	5.87%	3.42%	8.90%	3.22	6.73	2.10
Carcinoid	Adults	Overall	399	5.05%	1.66%	9.95%	12.86	68.90	0.58
		Europe	131	3.22%	0.75%	7.07%	0.86	0.00	−1.72
		North America	110	8.96%	0.70%	22.97%	3.83	73.92	−1.99
	Overall	Overall	2134	2.34%	1.74%	3.02%	3.36	0.00	3.31
		Asia	655	3.12%	1.91%	4.60%	0.07	0.00	−4.32
		Europe	397	2.04%	0.85%	3.70%	0.82	0.00	3.11
Colonic/Duodenal Tissue	Overall	Overall	2071	1.91%	0.75%	3.53%	20.43	65.74	5.29
		Asia	655	0.85%	0.00%	2.81%	1.95	48.62	4.30
		North America	1350	2.95%	0.63%	6.61%	14.72	72.83	6.12
	Pediatric	Overall	569	7.65%	3.20%	13.65%	33.81	79.30	2.94
		Asia	335	12.91%	2.59%	28.28%	27.13	88.94	1.60
		North America	150	4.14%	0.58%	10.05%	1.92	47.98	−2.11

## Data Availability

The data that support the findings of this study are available from the corresponding authors upon reasonable request.

## Supplementary Materials

The following supporting information can be downloaded at: https://www.mdpi.com/article/10.3390/jcm15103599/s1: Supplementary Table S1. Full search strategy; Supplementary Table S2. PRISMA 2020 checklist; Supplementary Table S3. Critical Appraisal Tool for Anatomical Meta-analysis (CATAM) assessment; Supplementary Material S4. The complete set of forest, funnel, and Doi plots and sensitivity analyses for each category; Supplementary Table S5. Characteristics of the studies included in this meta-analysis; Supplementary Material S6. AQUA assessment with detailed statistics.
