# Supplementary material for: Clinical and Anatomical Spectrum of Meckel’s Diverticulum: A Systematic Review and Meta-Analysis
Source: J Clin Med. 2026 May 8;15(10):3599. doi: 10.3390/jcm15103599 (PMC13207036; doi:10.3390/jcm15103599)
Supplement: Supplementary file 1 [file jcm-15-03599-s001.zip › Supplementary Table S1.pdf]

Database	Search String
PubMed	("Meckel Diverticulum"[MeSH] OR "Meckel's diverticulum"[Title/Abstract] OR "Meckel diverticulum"[Title/Abstract])
Scopus	("Meckel diverticulum" OR "Meckel's diverticulum")
Web of Science	("Meckel diverticulum" OR "Meckel's diverticulum")
ScienceDirect	Title, abstract or author-specified keywords: ("Meckel diverticulum" OR "Meckel's diverticulum")
SciELO	("Meckel diverticulum" OR "Meckel's diverticulum")
BIOSIS	("Meckel diverticulum" OR "Meckel's diverticulum")
Current Contents Connect	("Meckel diverticulum" OR "Meckel's diverticulum")
Korean Journal Database	("Meckel diverticulum" OR "Meckel's diverticulum")

Supplementary Table S1 | Full Search Strategy
