# Supplementary material for: Clinical and Anatomical Spectrum of Meckel’s Diverticulum: A Systematic Review and Meta-Analysis
Source: J Clin Med. 2026 May 8;15(10):3599. doi: 10.3390/jcm15103599 (PMC13207036; doi:10.3390/jcm15103599)
Supplement: Supplementary file 1 [file jcm-15-03599-s001.zip › Supplementary Table S5.pdf]

Study	Country, Continent	Patients	Patients with MD	Age group	Main Outcomes	AQUA				
						D1	D2	D3	D4	D5
Aarnio, P 2000	Finland, Europe	3758	71	Overall	Complication rates, Epidemiological data, Prevalence of MD, Clinical Manifestations	Low	Low	Low	Low	Low
Aktan, AO 1997	Turkey, Asia	2558	34	Overall	Epidemiological data, Prevalence of MD	Low	High	Low	Low	Low
Al Janabi, M. 2014	UK, Europe	73	73	Overall	Complication rates, Epidemiological data, Histopathology heterotopic tissue, Clinical Manifestations, Postoperative outcomes	High	High	Low	Low	Low
Albu, I 1993	Romania, Europe	878	11	Pediatric	Prevalence of MD	Low	Low	Low	Low	Low
Alemayehu, H 2014a	USA, North America	14	14	Pediatric	Complication rates, Epidemiological data, Clinical Manifestations, Postoperative outcomes	Low	Low	Low	Low	Low
Alemayehu, H 2014b	USA, North America	4338396	2389	Pediatric	Complication rates, Prevalence of MD, Clinical Manifestations	Low	Low	Low	Low	Low
Al-shawan, S 1996	Saudi Arabia, Asia	39	39	Pediatric	Complication rates, Epidemiological data, Histopathology heterotopic tissue, Clinical Manifestations, Postoperative outcomes	High	High	High	High	High
Arnold, JF 1997	USA, North America	58	58	Overall	Postoperative outcomes, Clinical Manifestations	Low	Low	Low	Low	Low
Aubrey, DA 1970	UK, Europe	66	66	Overall	Complication rates, Histopathology heterotopic tissue, Clinical Manifestations, Postoperative outcomes,	Low	Low	High	Low	High
Baeza, P 2022	Chile, South America	14	14	Overall	Epidemiological data, Postoperative outcomes, Morphological Data	High	Low	Low	Low	Low
Baltes, P 2023	Europe, Europe	69	69	Overall	Epidemiological data, Histopathology heterotopic tissue	Low	Low	Low	Low	Low
Bandi, A 2013	UK, Europe	39	39	Pediatric	Complication rates, Histopathology heterotopic tissue, Clinical Manifestations	Low	Low	Low	Low	Low
Bani-hani, KE 2004	Jordan, Asia	68	68	Overall	Complication rates, Epidemiological data, Histopathology heterotopic tissue, Clinical Manifestations, Postoperative outcomes, Morphological Data	High	Low	High	High	High
Bemelman, WA 1995	Netherlands, Europe	136	136	Overall	Complication rates, Epidemiological data, Histopathology heterotopic tissue, Clinical Manifestations, Postoperative outcomes	High	Low	Low	Low	Low
Benson, CD 1956	USA, North America	60	60	Pediatric	Histopathology heterotopic tissue, Postoperative outcomes	Low	High	High	Low	High
Berquist, TH 1976	USA, North America	100	8	Overall	Histopathology heterotopic tissue	High	High	High	High	High
Bindra, MHS 1997	India, Asia	25	25	Overall	Complication rates, Epidemiological data, Histopathology heterotopic tissue, Postoperative outcomes, Clinical Manifestations	High	Low	Low	Low	Low
Blanco, JM 1986	Spain, Europe	35	35	Overall	Complication rates, Clinical Manifestations, Postoperative outcomes	Low	Low	Low	Low	High
Brookes, VS 1954	UK, Europe	43	43	Pediatric	Epidemiological data, Histopathology heterotopic tissue, Postoperative outcomes	Low	Low	Low	Low	Low
Brungardt, JG 2020	USA, North America	506	506	Adult	Epidemiological data, Postoperative outcomes	Low	Low	Low	Low	Low
Burjonrappa, S 2014	USA, North America	22	22	Pediatric	Complication rates, Epidemiological data, Histopathology heterotopic tissue, Clinical Manifestations	Low	Low	Low	Low	Low
Can, M 2024	Turkey, Asia	139	139	Pediatric	Complication rates, Epidemiological data, Histopathology heterotopic tissue, Clinical Manifestations, Postoperative outcomes	Low	Low	Low	Low	Low
Canty, T 1975	USA, North America	60	60	Pediatric	Complication rates	Low	Low	High	High	Low

Carstensen, G 1983	Germany, Europe	155	155	Overall	Complication rates, Epidemiological data, Histopathology heterotopic tissue, Clinical Manifestations, Clinical Manifestations	Low	Low	Low	Low	Low
Celebl, S 2016	Turkey, Asia	137	137	Adult	Complication rates, Epidemiological data, Histopathology heterotopic tissue, Clinical Manifestations, Postoperative outcomes, Morphological Data	High	High	Low	Low	Low
Chaffin, L 1940	USA, North America	19	19	Pediatric	Complication rates, Epidemiological data, Clinical Manifestations, Postoperative outcomes	High	Low	Low	High	Low
Chan, KW 2008	China, Asia	20	20	Pediatric	Complication rates, Epidemiological data, Histopathology heterotopic tissue, Clinical Manifestations, Postoperative outcomes	Low	Low	Low	Low	Low
Chang, K 2021a	Taiwan, Asia	840	14	Adult	Epidemiological data, Histopathology heterotopic tissue, Prevalence of MD, Postoperative outcomes, Morphological Data	Low	Low	Low	Low	Low
Chang, YC 2021b	Taiwan, Asia	2453	2453	Overall	Complication rates, Epidemiological data, Postoperative outcomes, Clinical Manifestations	Low	Low	Low	Low	Low
Chen, Q 2018	China, Asia	286	286	Pediatric	Complication rates, Epidemiological data, Histopathology heterotopic tissue, Clinical Manifestations, Postoperative outcomes, Morphological Data	Low	Low	Low	Low	Low
Chou, JW 2021	Taiwan, Asia	55	55	Overall	Epidemiological data, Histopathology heterotopic tissue, Morphological Data	High	Low	Low	High	High
Cornell, GN 1954	USA, North America	6821	34	-	Prevalence of MD	Low	Low	Low	Low	Low
Cserni, G 1996	Hungary, Europe	140	140	Overall	Epidemiological data, Histopathology heterotopic tissue	Low	Low	Low	Low	Low
Cserni, G 1998	Hungary, Europe	166	166	Overall	Histopathology heterotopic tissue	High	High	High	Low	Low
Cullen, JJ 1994	USA, North America	145	145	Overall	Complication rates, Epidemiological data, Clinical Manifestations, Postoperative outcomes	Low	High	High	Low	Low
Dalinka, MK 1973	USA, North America	38	38	-	Complication rates, Clinical Manifestations	Low	High	High	High	High
Daneman, A 1998	Canada, North America	64	64	Pediatric	Epidemiological data	High	Low	Low	Low	High
Davidson, CL 1954	UK, Europe	5925	35	-	Prevalence of MD	Low	Low	Low	Low	Low
De Cothi, GA 1989	UK, Europe	69	69	-	Histopathology heterotopic tissue, Clinical Manifestations	Low	High	High	Low	High
Debartolo, HM 1976	USA, North America	190	190	Overall	Complication rates, Epidemiological data, Histopathology heterotopic tissue, Clinical Manifestations, Postoperative outcomes	Low	High	High	Low	High
Demartines, N 1992	Switzerland, Europe	91	91	Overall	Epidemiological data, Postoperative outcomes, Clinical Manifestations	High	Low	Low	High	Low
Demirel 2019, BD	Turkey, Asia	62	62	Pediatric	Epidemiological data, Histopathology heterotopic tissue	Low	Low	Low	Low	Low
Devi, GK 2022	Singapore, Asia	99	99	Pediatric	Complication rates, Epidemiological data, Histopathology heterotopic tissue, Clinical Manifestations	Low	Low	Low	Low	Low
Diamond, T 1985	UK, Europe	49	49	Overall	Complication rates, Epidemiological data, Histopathology heterotopic tissue, Postoperative outcomes, Clinical Manifestations	Low	Low	Low	Low	Low
Digiacomo, JC 1993	USA, North America	21	21	Overall	Complication rates, Epidemiological data, Histopathology heterotopic tissue, Postoperative outcomes, Clinical Manifestations	Low	Low	Low	Low	Low
Dixon, PM 1987	UK, Europe	14	14	Overall	Clinical Manifestations, Complication rates	Low	High	High	Low	Low
Dong, D 2020	India, Asia	15	15	Adult	Complication rates, Epidemiological data, Clinical Manifestations	Low	High	High	High	High
Duan, X 2015	China, Asia	55	55	Pediatric	Epidemiological data	Low	Low	Low	High	Low

Egan, TJ 1967	Ireland, Europe	18	18	Overall	Complication rates, Epidemiological data, Histopathology heterotopic tissue, Postoperative outcomes, Clinical Manifestations	High	High	High	High	Low
El-Maadawy, S 2021	Egypt, Africa	45	45	Pediatric	Epidemiological data, Histopathology heterotopic tissue	Low	Low	Low	Low	High
Ergun, O 2002	Turkey, Asia	30	30	Pediatric	Epidemiological data, Histopathology heterotopic tissue, Clinical Manifestations	High	High	High	High	Low
Erol, V 2013	Turkey, Asia	14	14	Adult	Complication rates, Epidemiological data, Histopathology heterotopic tissue, Postoperative outcomes	High	High	Low	High	Low
Esposito, C 2016	Europe 5 centers, Europe	1092	6	-	Prevalence of MD	Low	Low	Low	Low	Low
Everhart, MW 1940	USA, North America	14	14	Pediatric	Epidemiological data, Postoperative outcomes	Low	Low	High	High	Low
Ezekian, B 2019	USA, North America	148	148	Overall	Epidemiological data, Postoperative outcomes	High	Low	Low	Low	Low
Fa-si-oen, R 1999	Netherlands, Europe	27	27	Overall	Complication rates, Epidemiological data, Histopathology heterotopic tissue, Clinical Manifestations	High	Low	Low	Low	Low
Fich, A 1990	Israel, Asia	222	222	Overall	Histopathology heterotopic tissue	High	High	High	High	Low
Figarska-Czerniawska, I 2013	Poland, Europe	1021	61	Pediatric	Complication rates, Epidemiological data, Histopathology heterotopic tissue, Prevalence of MD, Clinical Manifestations	High	Low	Low	High	High
Finn 2001, LS	USA, North America	45	45	Pediatric	Histopathology heterotopic tissue	High	High	High	High	Low
Fonseca, S 2021	Portugal, Europe	47	47	Pediatric	Epidemiological data, Histopathology heterotopic tissue	High	Low	Low	Low	Low
Francis, A 2016	USA, North America	208	208	Pediatric	Epidemiological data, Histopathology heterotopic tissue	Low	Low	Low	Low	Low
McKay, R 2007	USA, North America	29	29	Overall	Epidemiological data, Histopathology heterotopic tissue, Clinical Manifestations	Low	Low	Low	Low	Low
Frederick, PL 1963	USA, North America	73	73	Pediatric	Complication rates, Histopathology heterotopic tissue, Clinical Manifestations	Low	High	High	Low	Low
Freedman, MA 1954	USA, North America	45	45	Pediatric	Complication rates, Histopathology heterotopic tissue, Clinical Manifestations	High	Low	Low	Low	Low
Fu, T 2021	China, Asia	28	28	Pediatric	Histopathology heterotopic tissue	Low	Low	Low	Low	Low
Garcia, C 2011	Spain, Europe	45	45	Overall	Postoperative outcomes	High	Low	Low	Low	Low
Gezer, HO 2016	Turkey, Asia	50	50	Pediatric	Complication rates, Epidemiological data, Histopathology heterotopic tissue, Clinical Manifestations	Low	Low	Low	Low	Low
Greenblatt, RB 1936	USA, North America	9000	18	Overall	Complication rates, Epidemiological data, Histopathology heterotopic tissue, Prevalence of MD, Clinical Manifestations	Low	Low	Low	Low	Low
Groebli, Y 2002	Switzerland, Europe	119	119	Adult	Complication rates, Histopathology heterotopic tissue, Postoperative outcomes, Clinical Manifestations	Low	Low	Low	Low	Low
Hashemian, H 1954	UK, Europe	18	18	Overall	Epidemiological data	High	Low	Low	Low	Low
He, Q 2013	China, Asia	783	74	Adult	Epidemiological data, Histopathology heterotopic tissue, Prevalence of MD	Low	Low	Low	High	High
Hernandez, JD 2023	Colombia, South America	27	27	Adult	Complication rates, Epidemiological data, Histopathology heterotopic tissue, Clinical Manifestations	Low	Low	Low	Low	Low
Howell, LM 1946	USA, North America	61	61	Overall	Epidemiological data, Histopathology heterotopic tissue	Low	Low	Low	Low	Low

Hu, J 2021	China, Asia	31	31	Pediatric	Epidemiological data, Histopathology heterotopic tissue, Morphological Data	Low	Low	Low	Low	Low
Huang, CC 2014	Taiwan, Asia	100	100	Pediatric	Epidemiological data, Histopathology heterotopic tissue, Morphological Data	Low	Low	Low	Low	Low
Hudson, H 1933	Turkey, Asia	90	90	Pediatric	Complication rates, Histopathology heterotopic tissue, Postoperative outcomes, Clinical Manifestations	High	High	High	High	High
Jackson, RH 1961	UK, Europe	82	82	Pediatric	Complication rates, Clinical Manifestations, Postoperative outcomes	Low	High	High	Low	High
Jay, GD 1950	USA, North America	103	103	Overall	Epidemiological data, Histopathology heterotopic tissue, Postoperative outcomes, Clinical Manifestations	Low	High	High	Low	Low
Johns, TNP 1959	USA, North America	143	143	Overall	Complication rates, Epidemiological data, Postoperative outcomes, Clinical Manifestations	Low	Low	Low	Low	Low
Jung, HS 2020	Korea, Asia	64	64	Overall	Epidemiological data, Histopathology heterotopic tissue, Postoperative outcomes, Clinical Manifestations	High	Low	Low	Low	Low
Kaihlanen, K 2024	USA, Europe	11	11	Pediatric	Epidemiological data, Histopathology heterotopic tissue	Low	High	High	Low	High
Kapral, W 1988	Austria, Europe	115	115	-	Clinical Manifestations	Low	Low	Low	Low	Low
Karabulut, R 2004	Turkey, Asia	29	29	Pediatric	Epidemiological data, Histopathology heterotopic tissue, Morphological Data, Clinical Manifestations	Low	Low	Low	Low	Low
Karaman, A 2009	Turkey, Asia	180	180	Pediatric	Histopathology heterotopic tissue, Postoperative outcomes, Clinical Manifestations	High	Low	Low	Low	High
Kashi, SH 1995	UK, Europe	35	35	-	Clinical Manifestations	Low	Low	Low	Low	Low
Kassem, H 2020	Egypt, Africa	17	17	Pediatric	Epidemiological data, Histopathology heterotopic tissue	Low	Low	Low	Low	Low
Kawamoto, S 2014	USA, North America	40	40	Overall	Complication rates, Histopathology heterotopic tissue, Morphological Data, Clinical Manifestations	Low	Low	Low	High	High
Khemekhema, R 2013	Tunis, Asia	40	40	Pediatric	Epidemiological data, Postoperative outcomes	Low	Low	Low	High	High
Kouraklis, G 2002	Greece, Europe	22	22	Adult	Complication rates, Epidemiological data, Clinical Manifestations	Low	Low	Low	Low	Low
Ksonz 2021, IV	Ukraine, Europe	183	183	Pediatric	Complication rates, Clinical Manifestations	Low	Low	Low	Low	Low
Kumar, R 2020	India, Asia	132	132	Pediatric	Epidemiological data, Histopathology heterotopic tissue, Postoperative outcomes, Clinical Manifestations	Low	Low	Low	Low	Low
Kusumoto, H 1992	Japan, Asia	776	776	Overall	Epidemiological data	Low	Low	High	Low	High
Lee, YA 2006	Korea, Asia	58	58	Pediatric	Epidemiological data, Histopathology heterotopic tissue, Morphological Data	Low	Low	Low	Low	Low
Leijonmarck, CE 1986	Sweden, Europe	260	260	Overall	Complication rates, Epidemiological data, Histopathology heterotopic tissue, Postoperative outcomes, Clinical Manifestations	Low	Low	High	Low	Low
Leonidas, JC 1974	USA, North America	13	13	Pediatric	Epidemiological data, Histopathology heterotopic tissue	Low	Low	Low	Low	High
Lin, X 2017	China, Asia	102	102	Pediatric	Complication rates, Epidemiological data, Histopathology heterotopic tissue, Clinical Manifestations	Low	Low	Low	Low	Low
Loh, JC 2013	Germany, Europe	29682	71	Overall	Complication rates, Epidemiological data, Histopathology heterotopic tissue, Prevalence of MD, Clinical Manifestations	Low	Low	Low	Low	Low
Lüdtke, FE 1989	Germany, Europe	84	84	Pediatric	Postoperative outcomes, Clinical Manifestations	Low	Low	Low	Low	Low
Mackey, WC 1983	USA, North America	153	153	Adult	Postoperative outcomes, Clinical Manifestations	Low	Low	Low	Low	Low
Mantas, D 2011	Greece, Europe	33	33	Adult	Complication rates, Clinical Manifestations	High	High	High	Low	Low

Martinez, N 2015	Paraguay, South America	14	14	Adult	Epidemiological data, Postoperative outcomes	Low	Low	Low	Low	Low
Matsagas, MI 1995	Greece, Europe	48	48	Overall	Complication rates, Epidemiological data, Histopathology heterotopic tissue, Morphological Data, Clinical Manifestations	Low	Low	Low	Low	Low
Mcdonald, JS 2022	USA, North America	76	76	Pediatric	Epidemiological data, Clinical Manifestations	Low	Low	High	Low	High
Mcparland, FA 1958	USA, North America	1809	21	-	Prevalence of MD	Low	Low	Low	Low	Low
Menezes, M 2008	Ireland, Europe	71	71	Pediatric	Complication rates, Epidemiological data, Histopathology heterotopic tissue, Clinical Manifestations	Low	High	High	Low	Low
Michas, CA 1975	USA, North America	35	35	Overall	Complication rates, Epidemiological data, Histopathology heterotopic tissue, Clinical Manifestations	High	Low	Low	Low	Low
Michel, ML 1955	USA, North America	100	100	Overall	Complication rates, Histopathology heterotopic tissue, Clinical Manifestations	Low	Low	Low	Low	Low
Migliaccio, AV 1948	USA, North America	50	50	Overall	Complication rates, Histopathology heterotopic tissue, Clinical Manifestations	Low	Low	Low	Low	Low
Mitchell, AW 1998	UK, Europe	16	16	Overall	Histopathology heterotopic tissue	High	Low	Low	High	High
Miyabara, S 1974	Japan, Asia	147	17	-	Prevalence of MD	Low	Low	Low	Low	Low
Mizutani, Y 2016	Japan, Asia	1747	33	Overall	Complication rates, Epidemiological data, Histopathology heterotopic tissue, Prevalence of MD, Clinical Manifestations, Morphological Data	Low	High	High	High	High
Mohammed, AA 2022	Iraq, Asia	70	70	Adult	Complication rates, Epidemiological data, Histopathology heterotopic tissue, Clinical Manifestations, Morphological Data	High	High	High	Low	High
Moore, T 1976	UK, Europe	50	50	Overall	Complication rates, Clinical Manifestations	High	High	High	Low	Low
Mora-Guzman, I 2018	Spain, Europe	66	66	Adult	Complication rates, Epidemiological data, Histopathology heterotopic tissue, Postoperative outcomes, Morphological Data, Clinical Manifestations	High	Low	Low	Low	Low
Morris, A 1989	New Zealand, Oceania	228	228	-	Histopathology heterotopic tissue	High	Low	Low	High	Low
Nissen, M 2022	Germany, Europe	62	62	Pediatric	Complication rates, Histopathology heterotopic tissue, Morphological Data, Clinical Manifestations	Low	High	High	Low	Low
Noel, WW 1940	USA, North America	25	25	Overall	Complication rates, Epidemiological data, Histopathology heterotopic tissue, Postoperative outcomes, Clinical Manifestations	Low	Low	High	Low	Low
Oguzkurt, P 2001	Turkey, Asia	74	74	Pediatric	Complication rates, Clinical Manifestations	Low	Low	Low	Low	Low
Olson, DE 2009	USA, North America	16	16	Pediatric	Epidemiological data	Low	Low	Low	Low	High
Onen, A 2003	Turkey, Asia	74	74	Pediatric	Complication rates, Epidemiological data, Postoperative outcomes, Clinical Manifestations	High	Low	Low	High	Low
Orszulok, J 1970	Ireland, Europe	27	27	Pediatric	Complication rates, Epidemiological data, Histopathology heterotopic tissue, Postoperative outcomes, Clinical Manifestations	High	High	Low	Low	Low
Ouangre, E 2015	Burkina Faso, Africa	11	11	Adult	Epidemiological data, Postoperative outcomes	Low	Low	Low	Low	Low
Palanivelu, C 2008	India, Asia	6700	20	Overall	Complication rates, Epidemiological data, Histopathology heterotopic tissue, Prevalence of MD, Clinical Manifestations	High	High	High	High	High

Park, JJ 2005	USA, North America	1476	1476	Overall	Complication rates, Epidemiological data, Histopathology heterotopic tissue, Postoperative outcomes, Clinical Manifestations	Low	Low	Low	Low	Low
Park, IK 2023	Korea, Asia	85	85	Overall	Complication rates, Epidemiological data, Clinical Manifestations, Postoperative outcomes, Morphological Data	High	Low	Low	Low	Low
Parvanescu, A 2018	France, Europe	37	37	Adult	Epidemiological data, Postoperative outcomes	Low	Low	Low	High	High
Passaro, E 1966	USA, North America	22	22	Adult	Epidemiological data, Clinical Manifestations	High	High	High	High	Low
Peoples, JB 1995	USA, North America	94	94	Adult	Postoperative outcomes, Clinical Manifestations	Low	Low	Low	Low	Low
Pickard, MA 1985	UK, Europe	28	28	Adult	Complication rates, Epidemiological data, Histopathology heterotopic tissue, Postoperative outcomes, Clinical Manifestations	Low	High	High	Low	Low
Pinero, A 2001	Spain, Europe	95	95	Overall	Complication rates, Epidemiological data, Histopathology heterotopic tissue, Postoperative outcomes, Morphological Data, Clinical Manifestations	Low	Low	Low	Low	Low
Popouis, G 1993	Greece, Europe	92	92	Pediatric	Histopathology heterotopic tissue, Clinical Manifestations	High	High	High	Low	Low
Priyadarshini, D 2023	India, Asia	18	18	Pediatric	Complication rates, Epidemiological data, Histopathology heterotopic tissue, Postoperative outcomes, Clinical Manifestations	Low	Low	Low	Low	Low
Ramesh, BH 2014	India, Asia	17	17	Pediatric	Epidemiological data, Histopathology heterotopic tissue	High	High	High	High	High
Ramesh, A 2017	India, Asia	45	45	Overall	Complication rates, Epidemiological data, Clinical Manifestations	Low	Low	Low	Low	Low
Rattan, K 2016	India, Asia	65	65	Pediatric	Epidemiological data, Histopathology heterotopic tissue, Postoperative outcomes	Low	Low	Low	Low	Low
Rerksuppaphol, S 2004	Australia, Oceania	37	37	Pediatric	Epidemiological data	Low	Low	Low	Low	Low
Rho, JH 2013	Korea, Asia	34	34	Pediatric	Epidemiological data, Histopathology heterotopic tissue, Postoperative outcomes, Morphological Data	High	Low	Low	Low	Low
Robinson, JR 2017	USA, North America	102	102	Pediatric	Complication rates, Epidemiological data, Histopathology heterotopic tissue, Postoperative outcomes, Clinical Manifestations	High	Low	Low	Low	Low
Ruscher, KA 2011	USA, North America	815	815	Pediatric	Complication rates, Epidemiological data, Clinical Manifestations	High	Low	High	Low	Low
Saiprasad, TR 2006	Singapore, Asia	36	36	Pediatric	Complication rates, Epidemiological data, Histopathology, heterotopic tissue, Postoperative outcomes, Clinical Manifestations	High	High	Low	Low	Low
Sakellaris, G 2011	Greece, Europe	45	45	Pediatric	Complication rates, Epidemiological data, Histopathology heterotopic tissue, Clinical Manifestations	Low	Low	High	Low	Low
Sancar, S 2015	Turkey, Asia	3429	57	Pediatric	Complication rates, Epidemiological data, Histopathology heterotopic tissue, Prevalence of MD, Postoperative outcomes, Clinical Manifestations	Low	Low	High	Low	Low
Shalaby, RY 2005	Egypt, Africa	1200	33	Pediatric	Complication rates, Epidemiological data, Histopathology heterotopic tissue, Prevalence of MD, Clinical Manifestations	High	Low	Low	Low	Low
Simms, MH 1980	UK, Europe	5919	171	-	Prevalence of MD	Low	Low	Low	Low	Low
Sinha, CK 2013	UK, Europe	183	183	Pediatric	Histopathology heterotopic tissue	Low	Low	Low	Low	Low
Sinopidsi, X 2019	Greece, Europe	146	146	Pediatric	Epidemiological data, Histopathology heterotopic tissue, Morphological Data	Low	Low	Low	High	Low
Skertich, NJ 2021	USA, North America	681	681	Pediatric	Postoperative outcomes	Low	Low	Low	Low	Low

Slinova, I 2018	Czech Republic, Europe	88	88	Pediatric	Epidemiological data, Histopathology heterotopic tissue, Morphological Data, Clinical Manifestations	High	Low	Low	High	Low
Sloan, RD 1954	Romania, Europe	10003	103	-	Prevalence of MD	Low	Low	Low	Low	Low
Soltero, MJ 1976	USA, North America	202	202	Overall	Clinical Manifestations, Complication rates	Low	Low	Low	Low	Low
Stanescu, GL 2014	Romania, Europe	44	44	Pediatric	Complication rates, Epidemiological data, Histopathology heterotopic tissue, Clinical Manifestations	Low	Low	Low	Low	Low
Steward, JH 1962	USA, North America	141	141	Overall	Complication rates, Epidemiological data, Histopathology heterotopic tissue, Postoperative outcomes, Clinical Manifestations	Low	Low	Low	Low	Low
Stone, PA 2004	USA, North America		47	Adult	Complication rates, Epidemiological data, Histopathology heterotopic tissue, Postoperative outcomes, Clinical Manifestations	Low	Low	Low	Low	Low
St-vil, D 1991	Canada, North America	164	164	Pediatric	Complication rates, Epidemiological data, Histopathology heterotopic tissue, Postoperative outcomes, Clinical Manifestations	Low	Low	Low	Low	Low
Tartaglia, D 2020	Italy, Europe	65	65	Adult	Complication rates, Epidemiological data, Histopathology heterotopic tissue, Morphological Data, Clinical Manifestations	High	High	Low	Low	Low
Tauro, L 2010	India, Asia	1332	15	Adult	Complication rates, Epidemiological data, Histopathology heterotopic tissue, Prevalence of MD, Postoperative outcomes, Clinical Manifestations	Low	Low	Low	Low	Low
Tree, K 2023	Australia, Oceania	160	160	Adult	Complication rates, Epidemiological data, Histopathology heterotopic tissue, Postoperative outcomes, Morphological Data, Clinical Manifestations	Low	Low	Low	Low	Low
Tseng, YY 2009	Taiwan, Asia	47	47	Pediatric	Complication rates, Epidemiological data, Histopathology heterotopic tissue, Clinical Manifestations	Low	Low	Low	Low	Low
Tuzun, A 2009	Turkey, Asia	21	21	Overall	Epidemiological data, Histopathology heterotopic tissue, Clinical Manifestations	High	Low	Low	High	High
Ueberrueck, T 2005	Germany, Europe	7927	233	Overall	Complication rates, Epidemiological data, Histopathology heterotopic tissue, Prevalence of MD, Postoperative outcomes, Clinical Manifestations	Low	High	Low	Low	Low
Ur rehman, I 2003	Pakisyan, Asia	63	63	-	Clinical Manifestations, Complication rates	Low	Low	Low	Low	Low
Vaabengaard, S 2020	Denmark, Europe	58	58	Pediatric	Epidemiological data, Histopathology heterotopic tissue, Postoperative outcomes	High	High	High	Low	Low
Varcoe, RL 2004	Australia, Oceania	77	77	Overall	Complication rates, Epidemiological data, Histopathology heterotopic tissue, Clinical Manifestations	Low	Low	High	Low	High
Wang, C 2023	China, Asia	40	40	Pediatric	Epidemiological data	Low	Low	Low	Low	Low
Wani, I 2010	India, Asia	14	14	Pediatric	Complication rates, Epidemiological data, Histopathology heterotopic tissue, Postoperative outcomes	Low	Low	High	Low	Low
Weistein, EC 1962	USA, North America	722	722	Adult	Complication rates, Histopathology heterotopic tissue, Clinical Manifestations	Low	Low	Low	Low	Low
Wu, H 2017	China, Asia	352	100	-	Histopathology heterotopic tissue	Low	Low	Low	Low	Low
Yamaguchi, M 1978	Japan, Asia	600	600	Overall	Epidemiological data, Histopathology heterotopic tissue	Low	Low	Low	Low	Low
Yan, L 2024	China, Asia	20	20	Pediatric	Complication rates, Epidemiological data, Histopathology heterotopic tissue, Clinical Manifestations, Postoperative outcomes	Low	High	High	Low	Low
Zhang, T 2023	China, Asia	20	20	Pediatric	Complication rates, Epidemiological data, Histopathology heterotopic tissue, Morphological Data, Clinical Manifestations	Low	Low	High	Low	Low

Zulfikaroglu, B 2008	Turkey, Asia	76	76	Adult	Complication rates, Epidemiological data Histopathology heterotopic tissue, Postoperative outcomes, Morphological Data, Clinical Manifestations	High	Low	High	Low	High

Supplementary Table S5 | Characteristic of the studies included in this meta-analysis.
