# Supplementary material for: Clinical and Anatomical Spectrum of Meckel’s Diverticulum: A Systematic Review and Meta-Analysis
Source: J Clin Med. 2026 May 8;15(10):3599. doi: 10.3390/jcm15103599 (PMC13207036; doi:10.3390/jcm15103599)
Supplement: Supplementary file 1 [file jcm-15-03599-s001.zip › Supplemetary Table S3.pdf]

Critical Appraisal Tool for Anatomical Meta-analysis (CATAM)

Domain	Criteria	Complete	Incomplete	Missing
Title	The title of the study should be succinct and include the word “meta-analysis.” (MA). The title should also contain the anatomical structure investigated in the study.	☒ 2 points	☐ 1 point	☐ 0 point
Abstract	The abstract should be well written and preferably structured (unless the journal guidelines require an unstructured abstract). Whether or not the abstract is structured, the following information should be included: (a) purpose of the MA, (b) databases used to retrieve the papers, (c) the tool used to assess the quality of the studies, (d) the guidelines used to conduct the MA, and (e) the data and pooled estimate if available	☒ 4 points	☐ 2 points	☐ 0 point
Introduction	The introduction should not be excessively long and the following information should be included: (a) description of the anatomical structure using the most recent nomenclature from Terminologia Anatomica, (b) literature review identifying the gap in the literature (epidemiological data may be included if relevant) and justification for why the MA was done, and (c) clear statement framing the research question.	☒ 6 points	☐ 3 points	☐ 0 point
Search Strategy	A statement that the Preferred Reporting Items for Systematic Reviews and Meta-Analyses (PRISMA) guidelines were followed should be in this subsection. The following information should also be included: (a) a list of all the databases searched (if hand searches were done, the details should be provided), (b) the start and end dates of the search should be disclosed, along with the languages, and (c) a list of all MeSH and non-	☒ 4 points	☐ 2 points	☐ 0 point

	MeSH terms in addition to the Boolean operators used			
Selection Criteria	The following information should be included: the study design (cadaveric, radiologic, surgical, etc.), subjects (cadavers or living humans), exposures, or outcomes. A list of the exclusion criteria that disqualified papers should also be mentioned. The number of researchers who applied the selection criteria should also be disclosed.	☒ 2 points	☐ 1 point	☐ 0 point
Data Extraction	The number of researchers who performed the data extraction should be mentioned along with any forms used to extract the data	☒ 2 points	☐ 1 point	☐ 0 point
Quality Assessment	The specific tool used to assess the quality of each included study in the MA should be mentioned and referenced. The Anatomical Quality Assessment (AQUA) tool is an excellent choice because it is specifically designed to be used for anatomical MAs	☒ 4 points	☐ 2 points	☐ 0 point
Statistical Analysis	The statistical software package (with version number) should be mentioned along with the name of the company that makes the software and its geographic location (city and state). The model used for the MA (e.g., fixed effects or random effects models) should also be disclosed. Homogeneity among the included studies should be disclosed using either the I^2 statistic or χ^2 statistic reporting	☒ 2 points	☐ 1 point	☐ 0 point
Search Results	The overall results of the search strategy (number of papers retained) should be mentioned along with the total number of subjects. These data must be shown in a bona fide flow chart that strictly adheres to the PRISMA guidelines	☒ 2 points	☐ 1 point	☐ 0 point
Characteristics of the included studies	The characteristics of the included studies should be presented in this section, preferably as a table	☒ 2 points	☐ 1 point	☐ 0 point
Outcomes	The main outcomes data should be presented in this section and should also be found as tables. Data should be	☒ 8 points	☐ 4 points	☐ 0 point

	presented in well-organized tables that include pooled means (when appropriate), 95% confidence intervals (CIs) and I^2 statistics. Most importantly, an anatomical illustration of the overall pooled data for the anatomical structure (and variations if appropriate) should be included			
Discussion	This section should include a summary and explanation of how the results relate to the research purpose. Researchers should expand on data from previous research and discuss how their meta-analytic data fit into the current paradigm. They should also explain what future studies on the topic should emphasize. Finally, they should include a thoughtful discussion of the limitations of their study	☒ 6 points	☐ 3 points	☐ 0 point
Conclusion	The conclusion section should summarize the purpose, results, and conclusions of the experiment. The implications for clinical practice should be stated	☒ 4 points	☐ 2 points	☐ 0 point
References	The references should be complete and every paper included in the MA should be listed here	☒ 2 points	☐ 1 point	☐ 0 point

Total Score: 50

CATAM Total Score Conversion Chart:

0–12 Poor

13–25 Fair

26–38 Good

39–50 Very good

Supplementary Table 3 | Assessment of the present Meta-analysis with Critical Appraisal Tool for Anatomical Meta-analysis (CATAM).
